# Data on fuel cell performance of Nafion® based hybrid composite membrane containing GO and dihydrogen phosphate functionalized ionic liquid at 70 °C under anhydrous condition

**DOI:** 10.1016/j.dib.2017.12.037

**Published:** 2017-12-20

**Authors:** Jatindranath Maiti, Nitul Kakati, Sung Pil Woo, Young Soo Yoon

**Affiliations:** a Department of Chemical Engineering, Gachon University, Gyeonggi-do 461-701, South Korea; b Department of Materials Science and Engineering, Yonsei University, Seoul 120-749, South Korea

## Abstract

This data provides the fuel cell performance of Nafion based hybrid membranes containing GO and dihydrogen phosphate functionalized ionic liquid (IL) at 70 °C under anhydrous condition. Readers are requested to go through the article entitled “Nafion® based hybrid composite membrane containing GO and dihydrogen phosphate functionalized ionic liquid for high temperature polymer electrolyte membrane fuel cell” (Maiti et al., 2017) [1] for further interpretation and discussion.

**Specifications Table**

**Table:** 

Subject area	*Chemistry*
More specific subject area	*Polymer Electrolyte Membrane*
Type of data	*Table, figure*
How data was acquired	*BioLogic EC-Lab, VSP-300*
Data format	*Analyzed*
Experimental factors	*Fuel cell performance was measured at 70* *°C under anhydrous condition*
Experimental features	*Unit fuel cell performance*
Data source location	Energy Materials Lab, Department of Chemical Engineering, Gachon University, Republic of Korea.
Data accessibility	*This article*

**Value of the data**

• Unit cell data on H_2_/O_2_ polymer electrolyte membrane fuel cell.
• Nafion based hybrid membrane containing GO and IL.
• Data on unit cell performance at 70 °C under anhydrous condition.

## 1 Data

**Fig. 1:**
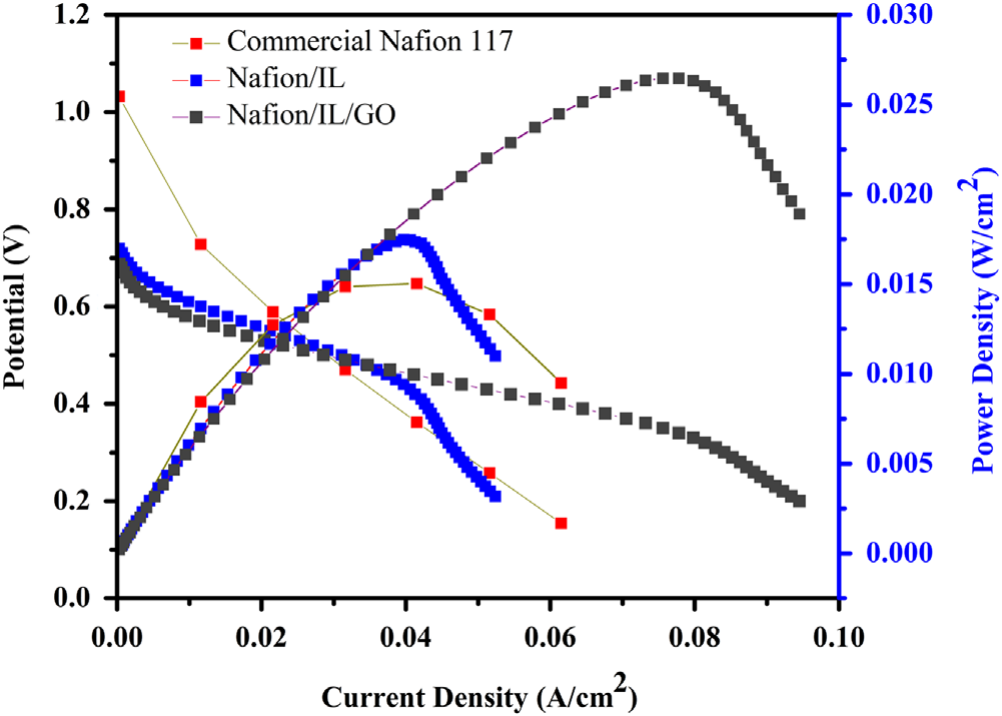
I–V and power density curves of membranes using H_2_ fuel at anode and O_2_ at cathode at 70 °C and 1 atm.

This dataset provides information on the performance of unit H_2_/O_2_ polymer electrolyte membrane fuel cells using commercial Nafion 117 membrane and Nafion based hybrid membranes containing GO and dihydrogen phosphate functionalized IL at 70 °C under anhydrous condition. Fig. 1 shows the I–V and power density curves obtained using the commercial Nafion 117 membrane and Nafion based hybrid membranes. The characteristic data like maximum power density and open circuit voltage of the unit cells using Nafion based membranes are tabulated in Table 1.

**Table 1:** Unit cell performance data of H_2_/O_2_ fuel cells using Nafion based membranes at 70 °C under anhydrous condition.

Sample	Maximum power density (W/cm^2^)	Open circuit potential (V)
Nafion	0.015	1.032
Nafion/IL	0.017	0.719
Nafion/IL/GO	0.026	0.687

## 2 Experimental design, materials and methods

2,3 dimethyl-1-butyl imidazolium dihydrogen phosphate (DMBuImH_2_PO_4_) was prepared from 1,2- Dimethyl imidazole and 1-bromobutane [1]. At first equal moles of 1,2- dimethyl imidazole and 1-bromobutane were mixed in a round bottom flask and stirred for 12 h at room temperature under argon atmosphere. The product was then washed three times with ethyl acetate and dried at 60 °C in a vacuum oven. The dried product was dissolved in acetonitrile and phosphoric acid was added drop-wise. After 48 h of reaction under argon atmosphere the solvent was evaporated using vacuum drying at 60 °C.

Solution casting process was used to prepare the Nafion/IL/GO and Nafion/IL membranes [1]. 0.015 g of GO was dispersed in DMF with 0.75 g of IL and stirred for 24 h. Then 0.75 g of Nafion 117 (vacuum dried at 60 °C for 12 h) was added to the above solution and further stirred for 24 h for a homogeneous solution. The solution was then poured onto a glass petri dish and placed in a vacuum oven at 110 °C for 4 h before peeling it off. Nafion/IL membrane was prepared similarly as above procedure by without adding GO. The thicknesses of membranes were kept at about 150 μm to 250 μm.

The membrane electrode assemblies (MEAs) were prepared by sandwiching the membrane between an anode and a cathode [1]. The anode and cathode were prepared by taking out 5 cm^2^ pieces of Pt/C coated carbon paper with a Pt loading of 1.6 mg cm^−2^. H_2_ in anode and O_2_ in cathode sides were supplied under anhydrous condition with a flow rate of 75 sccm and 150 sccm respectively. The polarization and power density curves of the unit cell were obtained at 70 °C after operating the cell for a activation period of 6 h.
