# Supplementary material for: Data on fuel cell performance of Nafion® based hybrid composite membrane containing GO and dihydrogen phosphate functionalized ionic liquid at 70 °C under anhydrous condition
Source: Data Brief. 2017 Dec 20;16:905–7. doi: 10.1016/j.dib.2017.12.037 (PMC5848106; doi:10.1016/j.dib.2017.12.037)
Supplement: Supplementary file 1 — Transparency document [file mmc1.pdf]

AUTHOR DECLARATION

We wish to draw the attention of the Editor to the following facts which may be considered as potential conflicts of interest and to significant financial contributions to this article entitled **"Data on fuel cell performance of Nafion® based hybrid composite membrane containing GO and dihydrogen phosphate functionalized ionic liquid at 70ºC under anhydrous condition"**. We wish to confirm that there are no known conflicts of interest associated with this publication.

We confirm that the manuscript has been read and approved by all named authors and that there are no other persons who satisfied the criteria for authorship but are not listed. We further confirm that the order of authors listed in the manuscript has been approved by all of us. We warrant that the article is the Authors' original work. We also warrant that the article has not received prior publication and is not under consideration for publication elsewhere. On behalf of all Co-Authors, the corresponding Author shall bear full responsibility for the submission.

We confirm that we have given due consideration to the protection of intellectual property associated with this work and that there are no impediments to publication, including the timing of publication, with respect to intellectual property. In so doing we confirm that we have followed the regulations of our institutions concerning intellectual property.

We understand that the Corresponding Author is the sole contact for the Editorial process (including Editorial Manager and direct communications with the office). He/she is responsible for communicating with the other authors about progress, submissions of revisions and final approval of proofs. We confirm that we have provided a current, correct email address (benedicto@gachon.ac.kr) which is accessible by the Corresponding Author and which has been configured to accept email from eesserver@eesmail.elsevier.com.

Signed by all authors as follows:

**Author List**	**Signature**
Prof. Young Soo Yoon (Corresponding Author)	
Dr. Jatindranath Maiti	
Dr. Nitul Kakati	
Mr. Sung Pil Woo	
